# Comparative Effectiveness of Peripheral Angioplasty Strategies for 1-Year Restenosis in Lower Limb Artery Disease: A Retrospective Single-Center Analysis

**DOI:** 10.3390/biomedicines13123100

**Published:** 2025-12-16

**Authors:** Ioannis Skalidis, Livio D’Angelo, Youcef Lounes, Francesca Sanguineti, Antoinette Neylon, Hakim Benamer, Benjamin Honton, Antoine Sauguet, Neila Sayah, Pietro Laforgia, Nicolas Amabile, Thomas Hovasse, Philippe Garot, Mariama Akodad, Stephane Champagne, Thierry Unterseeh

**Affiliations:** 1 Institut Cardiovasculaire Paris-Sud, Hôpital Jacques Cartier, 91300 Massy, France; 2 Department of Cardiology, HFR-Fribourg Kantonal Hospital and University, 1708 Fribourg, Switzerland; 3 Department of Cardiology, Clinique Pasteur, 31076 Toulouse, France

**Keywords:** peripheral artery disease, drug-coated balloon, restenosis, endovascular therapy, femoropopliteal angioplasty

## Abstract

**Background:** Optimal endovascular strategy for lower limb peripheral artery disease (PAD) remains debated, particularly regarding 1-year restenosis. **Aim:** To evaluate the association between drug-coated balloon (DCB)-based angioplasty and 1-year restenosis compared with stent-based and plain balloon strategies in a real-world PAD cohort. **Methods:** We performed a single-center retrospective analysis of 283 consecutive patients (mean age 67.5 ± 11.2 years, 79% male) undergoing lower limb angioplasty between 2010 and 2023. Patients were treated with one of five strategies: direct stent, pre-dilatation + stent, stent + post-dilatation, DCB ± bailout stent, or plain old balloon angioplasty (POBA). Restenosis at 12 months (≥50% diameter reduction on Doppler, CT angiography, or angiography) was the primary endpoint. Kaplan-Meier curves and multivariable Cox regression adjusted for clinical and lesion characteristics were used. The prespecified main comparison was DCB ± stent versus non-DCB strategies. **Results:** Overall, 1-year restenosis occurred in 81 patients (28.7%). Restenosis rates were 15.4% with DCB ± stent versus 34.2%, 29.8%, 31.5%, and 38.1% with direct stent, pre-dilatation + stent, stent + post-dilatation, and POBA, respectively (*p* = 0.004). In adjusted analysis, DCB ± stent was associated with a lower hazard of restenosis compared with direct stent (HR 0.52; 95% CI 0.31–0.87), whereas POBA was associated with a higher hazard versus DCB ± stent (HR 1.89; 95% CI 1.01–3.53). Periprocedural complication rates were low and similar across groups (overall 6.3%). **Conclusions:** In this real-world PAD cohort, DCB-based angioplasty was independently associated with lower 1-year restenosis compared with stent-based and plain balloon strategies, without an excess of procedural complications. Given the observational design and lesion-driven strategy selection, these findings should be interpreted as hypothesis-generating but support broader use of DCB in appropriately selected lesions.

## 1. Introduction

Peripheral artery disease (PAD) affects over 200 million individuals globally and represents a major source of cardiovascular morbidity and functional impairment, particularly among aging populations [1]. In patients with chronic limb-threatening ischemia, endovascular revascularization is guideline-supported as an effective revascularization strategy, whereas in intermittent claudication, supervised exercise therapy and risk-factor optimization represent first-line therapy. Revascularization is reserved for patients with lifestyle-limiting symptoms that persist despite optimal conservative management [2].

Historically, plain balloon angioplasty (POBA) was widely used but suffered from high rates of elastic recoil and restenosis, especially in long, calcified, or ostial lesions [3]. The introduction of nitinol stents offered mechanical scaffolding to prevent recoil and flow-limiting dissection, improving early outcomes; however, in-stent restenosis (ISR) remained a persistent limitation [4].

More recently, drug-coated balloons (DCBs) have been developed to deliver antiproliferative agents—typically paclitaxel—without leaving a permanent implant. These devices offer the theoretical advantage of reducing neointimal hyperplasia while avoiding the long-term risks associated with stent placement. Randomized controlled trials such as the IN.PACT SFA and PACIFIER trials demonstrated superior patency rates and lower target lesion revascularization (TLR) compared to standard balloon angioplasty [5,6].

Real-world data have since confirmed the effectiveness of DCBs in complex lesion subsets, with reported 1-year primary patency rates exceeding 75% in contemporary registries [7]. Furthermore, comparative studies have begun to evaluate different DCB dosing strategies, suggesting non-inferiority of low-dose versus high-dose paclitaxel formulations in terms of restenosis and safety [8,9]. A recent systematic review of randomized trials supports the long-term efficacy and safety of paclitaxel-coated balloons in the femoropopliteal segment [10].

Despite these advances, real-world decision-making in peripheral interventions remains highly heterogeneous. Operators choose between direct stenting, various stent optimization techniques, DCB ± bailout stenting, or POBA according to lesion morphology, vessel size, and procedural complexity rather than through standardized algorithms. As a result, comparative data on how these routinely used strategies influence restenosis risk in unselected PAD populations remain limited.

The aim of this study was therefore to evaluate the association between DCB-based angioplasty and 1-year restenosis, compared with stent-based and plain balloon strategies, in a real-world cohort of patients undergoing lower limb endovascular revascularization for symptomatic PAD.

## 2. Materials and Methods

### 2.1. Study Design and Population

We conducted a single-center retrospective cohort study including all consecutive patients who underwent endovascular revascularization for symptomatic lower limb PAD between January 2010 and December 2023 at Claude Galien Hospital, Institut Cardiovasculaire Paris-Sud, France. Patients were eligible if they received percutaneous transluminal angioplasty (PTA) with at least one of the following strategies: (1) direct stent implantation, (2) pre-dilatation followed by stenting, (3) stenting with post-dilatation, (4) DCB with or without bailout stenting, or (5) POBA without stenting or drug application.

Stent-based strategy subcategories were defined according to whether lesion predilatation and/or post-dilatation were documented as separate procedural steps in the institutional registry. Because these steps were not systematically captured across the entire study period, their absence in the database does not necessarily indicate that they were not performed. Bailout stenting was defined as unplanned stent implantation following balloon angioplasty due to suboptimal angiographic results, including significant residual stenosis or flow-limiting dissection.

**Figure 1:**
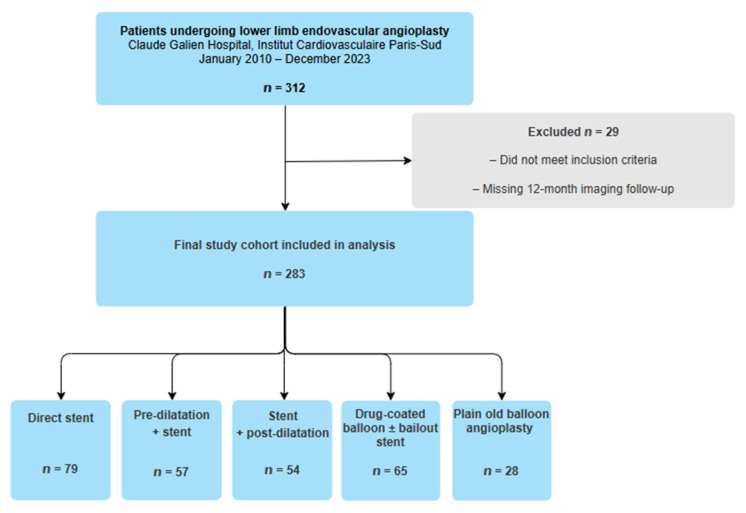
Flowchart of the Study.

Patients were excluded if they underwent surgical bypass or lacked follow-up imaging data. All procedures were performed by four experienced interventionalists using standard femoral or radial access and fluoroscopic guidance, following institutional protocols. The choice of angioplasty strategy was left to the operator and was primarily guided by lesion morphology, vessel size, and device availability. Thus, the present analysis reflects consistent practice patterns within a single medium-volume center rather than sporadic procedures performed by low-experience operators. The patient selection process is summarized in Figure 1.

### 2.2. Data Collection

Demographic data, cardiovascular risk factors (e.g., diabetes mellitus, smoking status, chronic kidney disease), lesion characteristics (length, diameter, calcification, chronic total occlusion), and procedural details (device type, balloon/stent characteristics) were prospectively recorded in a dedicated institutional database. In DCB-treated lesions, lesion preparation was performed according to standard clinical practice; however, predilatation was recorded only when documented as a separate procedural step, which may have resulted in underreporting of predilatation rates in the registry. Imaging follow-up at approximately 12 months was performed predominantly using duplex Doppler ultrasound as part of routine surveillance. Computed tomography angiography (CTA) or conventional angiography was reserved for selected cases, such as recurrent symptoms, inconclusive ultrasound findings, or when reintervention was being considered. The choice of follow-up imaging modality was based on clinical considerations and was not determined by the initial endovascular treatment strategy.

### 2.3. Study Endpoints

The primary endpoint was binary restenosis at 12 months, defined as ≥50% luminal diameter reduction by imaging. Secondary endpoints included procedural success, defined as residual stenosis < 30% without persistent flow-limiting dissection at the end of the procedure, and occurrence of access-site or periprocedural complications (bleeding or pseudoaneurysm). Persistent flow-limiting dissections were recorded only when present at the end of the procedure or when requiring additional unplanned intervention.

### 2.4. Statistical Analysis

Continuous variables are presented as mean ± standard deviation, and categorical variables as counts and percentages. Comparisons between angioplasty strategy groups were performed using analysis of variance (ANOVA) for continuous variables and the chi-square test for categorical variables, as appropriate. Restenosis-free survival was assessed using Kaplan-Meier estimates and compared using the log-rank test. Multivariable Cox proportional hazards models were constructed with careful consideration of model parsimony to avoid overfitting. Age, sex, and diabetes mellitus were selected a priori as key clinical covariates based on biological plausibility and data completeness. Lesion-related variables, including lesion length, reference vessel diameter, and calcification severity, were included based on their established association with restenosis risk and consistent availability in the dataset. Adjusted hazard ratios (HRs) are reported with 95% confidence intervals (CIs).

## 3. Results

### 3.1. Baseline Characteristics

A total of 283 patients were included (mean age 67.7 ± 11.0 years; 79% male). The distribution of angioplasty strategies was: direct stent in 79 patients (27.9%), pre-dilatation + stent in 57 (20.1%), stent + post-dilatation in 54 (19.1%), DCB ± stent in 65 (23.0%), and POBA in 28 (9.9%). Baseline demographic characteristics and cardiovascular risk factors were well balanced across groups, with no significant differences in age, sex, diabetes mellitus, hypertension, dyslipidemia, smoking history, or chronic kidney disease (all *p* > 0.05) (Table 1). Baseline clinical severity was assessed using Rutherford classification.

Lesion characteristics differed meaningfully between strategies. Lesion length was shortest in the DCB ± stent group (64.2 ± 22.5 mm) compared with the stent-based and POBA groups (72.8–81.9 mm; *p* = 0.04). Moderate-to-severe calcification was also less frequent in the DCB ± stent group (21.5% vs. 29.8–36.0%; *p* = 0.03). There were no significant differences in reference vessel diameter (*p* = 0.12) or prevalence of chronic total occlusion (*p* = 0.27).

### 3.2. Procedural Characteristics and Periprocedural Outcomes

Procedural steps reflected the predefined strategy categories (Table 2). Pre-dilatation was performed in all patients in the pre-dilatation + stent group and in a minority of the other groups (*p* < 0.001). Post-dilatation occurred in all patients in the stent + post-dilatation group and infrequently elsewhere (*p* < 0.001). Bailout stenting was required in 18.5% of DCB ± stent procedures.

Overall procedural success was high (91.5%), with significant variation across strategies (*p* = 0.02). The highest success rate was observed in the DCB ± stent group (96.8%), while the lowest was in the POBA group (82.1%). Periprocedural complications were uncommon (6.3%) and showed no significant differences between strategies (*p* = 0.62). The most frequent events were access-site hematoma (2.5–7.1%) and pseudoaneurysm (0–3.6%). Persistent flow-limiting dissection (1.5–7.1%) was infrequent and was recorded only when present at the end of the procedure or requiring additional unplanned intervention. The distribution of lesion segments across treatment strategies is also shown in Supplementary Materials Table S1.

### 3.3. Primary Endpoint: 12-Month Restenosis

Restenosis at 12 months occurred in 81 patients (28.7%). Restenosis rates differed significantly across strategies (*p* = 0.004), with the lowest rate in the DCB ± stent group (15.4%) and the highest in the POBA group (38.1%). Stent-based strategies demonstrated intermediate rates (29.8–34.2%) (Table 3).

Kaplan-Meier estimates showed that restenosis-free survival at 12 months was significantly higher with DCB ± stent (84.6%) than with stent-based strategies (65.1–68.2%) or POBA (61.9%), with an overall log-rank *p* = 0.002.

### 3.4. Adjusted Outcomes

**Figure 2:**
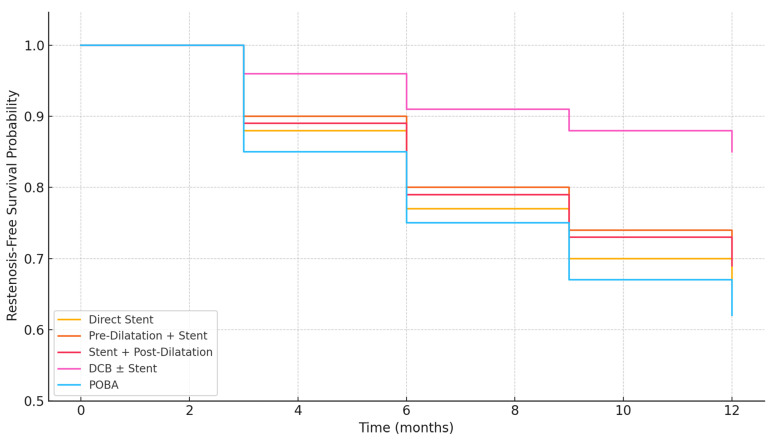
Kaplan-Meier Analysis of 1-Year Restenosis-Free Survival According to Angioplasty Strategy. Restenosis-free survival was significantly higher in patients treated with drug-coated balloon (DCB) ± stent compared to those receiving direct stenting, pre-dilatation + stent, stent + post-dilatation, or plain old balloon angioplasty (POBA) (log-rank *p* = 0.002). The DCB ± stent group demonstrated the most favorable 12-month patency, while POBA and direct stenting had the highest restenosis rates. DCB, drug-coated balloon; POBA, plain old balloon angioplasty.

**Figure 3:**
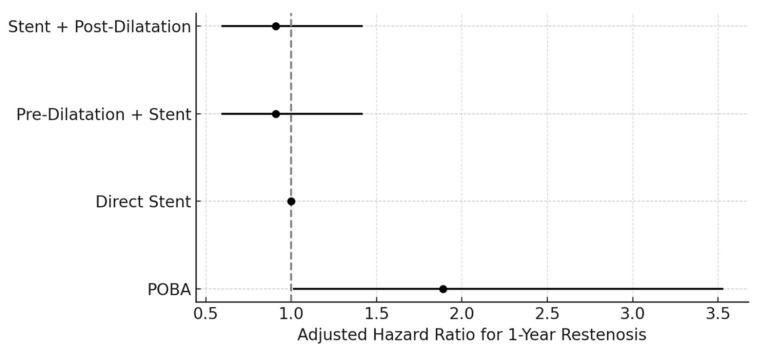
Adjusted Hazard Ratios for 1-Year Restenosis According to Angioplasty Strategy. Multivariable Cox regression analysis comparing peripheral angioplasty strategies using DCB ± stent as the reference. Plain old balloon angioplasty (POBA) was associated with a significantly increased hazard for 1-year restenosis (HR 1.89, 95% CI: 1.01–3.53). No significant differences were found between stent-based strategies and the reference group. The dashed vertical line indicates the null value (HR = 1.0). Error bars represent 95% confidence intervals. DCB, drug-coated balloon; POBA, plain old balloon angioplasty.

In the multivariable Cox regression model using direct stent as the reference, the DCB ± stent strategy was independently associated with a reduced risk of restenosis at 12 months (adjusted HR 0.52; 95% CI 0.31–0.87). Neither pre-dilatation + stent nor stent + post-dilatation differed significantly from direct stent (both adjusted HR 0.91; 95% CI 0.59–1.42) (Figure 2). In the complementary model using DCB ± stent as the reference, POBA was associated with a higher hazard of restenosis (adjusted HR 1.89; 95% CI 1.01–3.53), while both stent-based strategies demonstrated no significant difference from DCB (Figure 3).

## 4. Discussion

In this real-world cohort of 283 patients undergoing endovascular revascularization for symptomatic PAD, we observed marked differences in 12-month restenosis between commonly used angioplasty strategies. The DCB ± stent group demonstrated the lowest restenosis rate at 15.4%, compared with 29.8–34.2% in the three stent-based strategies and 38.1% with POBA. These findings remained consistent after adjustment for key clinical and anatomical variables, with DCB therapy associated with a 48% lower hazard of restenosis relative to direct stenting (adjusted HR 0.52; 95% CI 0.31–0.87). Conversely, POBA was associated with nearly a two-fold higher hazard of restenosis compared with the DCB strategy (adjusted HR 1.89; 95% CI 1.01–3.53).

These observations align with the established evidence base supporting paclitaxel-coated balloon therapy. Randomized trials such as IN.PACT SFA and PACIFIER [5,6] demonstrated higher primary patency and lower target lesion revascularization with DCB therapy compared with standard PTA. Reported 1-year restenosis or loss-of-patency rates in DCB arms of major trials typically range from 12% to 20%, a range that is highly consistent with the 15.4% observed in our cohort. Likewise, long-term outcomes from pooled analyses and meta-analyses [7,8,9,10] have shown sustained reduction in neointimal hyperplasia with antiproliferative drug delivery.

However, unlike previous randomized studies that compare DCB therapy to a single control device, our analysis evaluated five distinct operator-selected strategies, reflecting the heterogeneity of routine clinical practice. This design allowed comparison not only between DCB and POBA, but also among commonly used stent-based approaches. The similar restenosis rates observed across direct stenting, pre-dilatation + stenting, and stent + post-dilatation (29.8–34.2%) suggest that, in this population, mechanical optimization maneuvers alone may have limited ability to meaningfully modify long-term vessel healing in the absence of drug delivery.

Procedural success exceeded 89% across all stent-based strategies and was highest in the DCB ± stent group (96.8%), indicating that DCB use did not compromise acute technical success. Periprocedural complications were infrequent across all strategies (6.3% overall), reinforcing the procedural safety of the approaches examined. Although lesions treated with DCB were somewhat shorter, the magnitude and consistency of benefit across both unadjusted and adjusted analyses suggest that this difference alone is unlikely to fully explain the observed outcomes.

Overall, these findings support the clinical relevance of antiproliferative therapy in optimizing long-term patency following peripheral angioplasty, while highlighting the limited incremental effect of stent optimization techniques on restenosis in real-world practice.

This study has several limitations. First, its retrospective, observational design introduces the potential for selection bias, as strategy choice was operator-dependent and influenced by lesion characteristics and procedural complexity. Although multivariable adjustment was performed, unmeasured confounding cannot be excluded. Second, the analysis reflects the experience of a single medium-volume center, which may limit external generalizability. Practice patterns, device selection, and thresholds for bailout stenting may vary across institutions, particularly in higher-volume centers with broader access to advanced technologies. Third, the sample size, particularly for the POBA group (*n* = 28), is modest. While statistical significance was reached for the primary comparisons, the study may be underpowered to detect smaller differences between individual stent-based techniques or to support detailed subgroup analyses. Fourth, although all patients underwent imaging follow-up at 12 months, different modalities (duplex ultrasound, CTA, or angiography) were used. These modalities have differing sensitivities and specificities for detecting ≥50% restenosis. While the choice of imaging was driven by clinical indications rather than treatment strategy, this heterogeneity may have introduced some degree of restenosis misclassification. Detailed data on post-procedural antiplatelet and anticoagulant therapy were not systematically available and could therefore not be included. Although all procedures were performed by experienced operators, the overall annual volume of femoropopliteal and infrapopliteal interventions was modest, and outcomes from very high-volume centers may differ. Finally, procedural variables such as balloon inflation pressure, stent expansion indices, and plaque composition were not systematically recorded. These technical factors may affect restenosis risk, but could not be incorporated into the adjusted models.

## 5. Conclusions

In this real-world single-center cohort of patients undergoing endovascular treatment for symptomatic PAD, DCB-based angioplasty was associated with a lower risk of restenosis compared with stent-based strategies and plain balloon angioplasty. Stent optimization techniques, including pre- and post-dilatation, did not meaningfully alter long-term patency within this population. These findings support the role of drug-coated balloon therapy as an effective strategy when anatomically feasible, while recognizing that individual lesion characteristics and procedural requirements may still necessitate stent deployment. Given the retrospective nature and single-center setting of this study, confirmation from larger, multicenter investigations is needed to clarify optimal treatment selection and to guide evidence-based decision-making in contemporary peripheral interventions.

## Figures and Tables

**Table 1:** Baseline Demographic, Clinical, and Lesion Characteristics by Angioplasty Strategy.

Variable	Direct Stent	Pre-Dilatation + Stent	Stent + Post-Dilatation	DCB ± Stent	POBA	*p*-Value
Number of patients	79	57	54	65	28	-
Age (years)	68.1 ± 10.9	67.4 ± 10.3	66.9 ± 11.5	67.2 ± 10.7	68.7 ± 11.8	0.63
Male sex (%)	77.2%	78.9%	79.6%	80.0%	78.6%	0.92
Diabetes mellitus (%)	45.6%	43.9%	42.6%	41.5%	46.4%	0.84
Hypertension (%)	72.2%	75.4%	70.4%	74.6%	73.2%	0.77
Dyslipidemia (%)	63.3%	66.7%	61.1%	64.6%	67.9%	0.81
Smoking (%)	60.8%	63.2%	59.3%	62.2%	64.3%	0.88
Chronic kidney disease (%)	28.0%	26.3%	27.8%	25.0%	28.6%	0.79
Lesion length (mm)	74.3 ± 24.6	72.8 ± 22.1	76.1 ± 23.9	64.2 ± 22.5	81.9 ± 27.3	0.04
Vessel diameter (mm)	4.8 ± 0.7	4.9 ± 0.6	4.7 ± 0.8	4.6 ± 0.7	4.5 ± 0.6	0.12
Calcification (moderate/severe) (%)	31.6%	29.8%	33.3%	21.5%	36.0%	0.03
Chronic total occlusion (%)	25.3%	22.8%	24.1%	20.0%	26.8%	0.27
Rutherford class 1–3, %	73.4	75.4	72.2	78.5	71.4	0.68
Rutherford class 4–6, %	26.6	24.6	27.8	21.5	28.6	0.68

Values are expressed as mean ± standard deviation or percentage, as appropriate. DCB, drug-coated balloon; POBA, plain old balloon angioplasty; mm, millimeter.

**Table 2:** Procedural Characteristics and Immediate Outcomes by Angioplasty Strategy.

Variable	Direct Stent (*n* = 79)	Pre-Dilatation + Stent (*n* = 57)	Stent + Post-Dilatation (*n* = 54)	DCB ± Stent (*n* = 65)	POBA (*n* = 28)	*p*-Value
Pre-dilatation performed (%)	12.7%	100%	8.3%	26.2%	21.4%	<0.001
Post-dilatation performed (%)	15.2%	10.5%	100%	20.0%	10.7%	<0.001
Bailout stenting (for DCB) (%)	–	–	–	18.5%	–	–
Procedural success (%)	91.1%	89.5%	90.7%	96.8%	82.1%	0.02
Access-site hematoma (%)	2.5%	1.8%	3.7%	1.5%	7.1%	0.34
Pseudoaneurysm (%)	1.3%	1.8%	0%	1.5%	3.6%	0.52
Persistent flow-limiting dissection (%)	2.5%	3.5%	1.9%	1.5%	7.1%	0.28
Any complication (%)	6.3%	7.0%	5.6%	4.6%	14.3%	0.62

DCB, drug-coated balloon; POBA, plain old balloon angioplasty. Values are reported as percentages unless otherwise specified. Procedural success was defined as <30% residual stenosis without major complications. Predilatation was defined as a separately documented lesion preparation step prior to the definitive treatment strategy. In DCB-treated le-sions, lesion preparation was routinely performed; however, predilatation was not always captured as a distinct variable in the registry.

**Table 3:** One-year restenosis outcomes and adjusted hazard ratios according to angioplasty strategy.

Variable	Direct Stent (*n* = 79)	Pre-Dilatation + Stent (*n* = 57)	Stent + Post-Dilatation (*n* = 54)	DCB ± Stent (*n* = 65)	POBA (*n* = 28)	*p*-Value
Restenosis at 12 months (%)	34.2%	29.8%	31.5%	15.4%	38.1%	0.004
Restenosis-free survival (Kaplan-Meier, %)	65.1%	68.2%	67.4%	84.6%	61.9%	0.002
Adjusted HR for restenosis (95% CI)	Reference	0.91 (0.59–1.42)	0.91 (0.59–1.42)	0.52 (0.31–0.87)	1.89 (1.01–3.53)	-
Procedural success (%)	91.1%	89.5%	90.7%	96.8%	82.1%	0.02

Values for restenosis, restenosis-free survival, procedural success, and complication rates are expressed as percentages. DCB, drug-coated balloon; POBA, plain old balloon angioplasty; HR, hazard ratio; CI, confidence interval.

## Data Availability

The data presented in this study are availavle on request from the corresponding author due to privacy reasons.

## Supplementary Materials

The following supporting information can be downloaded at: https://www.mdpi.com/article/10.3390/biomedicines13123100/s1, Table S1: Distribution of lesion segments across treatment strategies (baseline anatomical distribution).

## Abbreviations

The following abbreviations are used in this manuscript:

ANOVA	Analysis of Variance
CI	Confidence Interval
CKD	Chronic Kidney Disease
CTA	Computed Tomography Angiography
CTO	Chronic Total Occlusion
DCB	Drug-Coated Balloon
HR	Hazard Ratio
ISR	In-Stent Restenosis
PAD	Peripheral Artery Disease
POBA	Plain Old Balloon Angioplasty
PTA	Percutaneous Transluminal Angioplasty
SD	Standard Deviation
TLR	Target Lesion Revascularization

## References

[1] Fowkes F.G.R., Rudan D., Rudan I., Aboyans V., Denenberg J.O., McDermott M.M., Norman P.E., Sampson U.K., Williams L.J., Mensah G.A. (2013). Comparison of global estimates of prevalence and risk factors for peripheral artery disease in 2000 and 2010: A systematic review and analysis. Lancet.

[2] Conte M.S., Bradbury A.W., Kolh P., White J.V., Dick F., Fitridge R., Mills J.L., Ricco J.-B., Suresh K.R., Murad M.H. (2019). Global vascular guidelines on the management of chronic limb-threatening ischemia. J. Vasc. Surg..

[3] Krankenberg H., Schlüter M., Steinkamp H. (2008). Nitinol Stent Implantation Versus Percutaneous Transluminal Angioplasty in Superficial Femoral Artery Lesions up to 10 cm in Length: The Femoral Artery Stenting Trial (FAST). J. Vasc. Surg..

[4] Laird J.R., Katzen B.T., Scheinert D., Lammer J., Carpenter J., Buchbinder M., Dave R., Ansel G., Lansky A., Cristea E. (2010). Nitinol Stent Implantation Versus Balloon Angioplasty for Lesions in the Superficial Femoral Artery and Proximal Popliteal Artery. Circ. Cardiovasc. Interv..

[5] Tepe G., Laird J., Schneider P., Brodmann M., Krishnan P., Micari A., Metzger C., Scheinert D., Zeller T., Cohen D.J. (2015). Drug-Coated Balloon Versus Standard Percutaneous Transluminal Angioplasty for the Treatment of Superficial Femoral and Popliteal Peripheral Artery Disease. Circulation.

[6] Werk M., Albrecht T., Meyer D.-R., Ahmed M.N., Behne A., Dietz U., Eschenbach G., Hartmann H., Lange C., Schnorr B. (2012). Paclitaxel-Coated Balloons Reduce Restenosis After Femoro-Popliteal Angioplasty. Circ. Cardiovasc. Interv..

[7] Kim H.J., Hwang D., Yun W.-S., Huh S., Kim H.-K. (2024). Effectiveness of Atherectomy and Drug-Coated Balloon Angioplasty in Femoropopliteal Disease: A Comprehensive Outcome Study. Vasc. Spéc. Int..

[8] Nakama T., Takahara M., Iwata Y., Suzuki K., Tobita K., Hayakawa N., Horie K., Mori S., Obunai K., Ohki T. (2023). Low-Dose vs High-Dose Drug-Coated Balloon for Symptomatic Femoropopliteal Artery Disease. JACC Cardiovasc. Interv..

[9] Steiner S., Schmidt A., Zeller T., Tepe G., Thieme M., Maiwald L., Schröder H., Euringer W., Popescu C., Brechtel K. (2022). Low-Dose vs High-Dose Paclitaxel-Coated Balloons for Femoropopliteal Lesions. JACC Cardiovasc. Interv..

[10] D’Oria M., Mastrorilli D., Secemsky E., Behrendt C.-A., Veraldi G., DeMartino R., Mani K., Budtz-Lilly J., Scali S., Saab F. (2023). Robustness of Longitudinal Safety and Efficacy After Paclitaxel-Based Endovascular Therapy for Treatment of Femoro-Popliteal Artery Occlusive Disease: An Updated Systematic Review and Meta-Analysis of Randomized Controlled Trials. Ann. Vasc. Surg..

