# Supplementary material for: Comparative Effectiveness of Peripheral Angioplasty Strategies for 1-Year Restenosis in Lower Limb Artery Disease: A Retrospective Single-Center Analysis
Source: Biomedicines. 2025 Dec 16;13(12):3100. doi: 10.3390/biomedicines13123100 (PMC12730210; doi:10.3390/biomedicines13123100)
Supplement: Supplementary file 1 [file biomedicines-13-03100-s001.zip › biomedicines-4037466-supplementary.pdf]

Supplementary Table S1. Distribution of lesion segments across treatment strategies (baseline anatomical distribution). Values represent the percentage of patients within each angioplasty strategy group with lesions located in the specified arterial segment. Percentages are reported descriptively and are not restenosis rates.

Segment	Direct Stent	Pre-Dilatation + Stent	Stent + Post-Dilatation	DCB ± Stent	POBA
Superficial femoral artery (SFA)	18.9%	20.4%	17.6%	14.7%	25.0%
Popliteal artery	21.7%	23.5%	20.0%	18.4%	28.6%
Infrapopliteal arteries	28.6%	31.3%	29.4%	24.1%	38.5%
